# The transverse facial artery anatomy: Implications for plastic surgery procedures

**DOI:** 10.1371/journal.pone.0211974

**Published:** 2019-02-07

**Authors:** Mateusz Koziej, Jakub Polak, Jakub Wnuk, Marek Trybus, Jerzy Walocha, Anna Chrapusta, Paweł Brzegowy, Ewa Mizia, Tadeusz Popiela, Mateusz Hołda

**Affiliations:** 1 Department of Anatomy, Jagiellonian University Medical College, Kraków, Poland; 2 The Malopolska Center for Burns and Plastic Surgery; The Ludwik Rydygier Hospital, Krakow, Poland; 3 Department of Radiology, Jagiellonian University Medical College, Kraków, Poland; 4 Second Department of General Surgery, Jagiellonian University Medical College, Kraków, Poland; University Magna Graecia of Catanzaro, ITALY

## Abstract

**Background:**

The transverse facial artery (TFA) perfuses the lateral face. Knowledge of topographical anatomy of the lateral face is crucial for safe procedural performance in aesthetic and plastic surgery, especially the face lift flap and face transplant. The aim of the present study was to assess detailed TFA morphometrical features.

**Patients and methods:**

One-hundred computed tomography head angiographies were analyzed. TFA numbers and origins were recorded bilaterally (200 cases). TFA diameters and lengths in addition to their positions in relation to neighboring vessels and the zygomatic arches were measured.

**Results:**

TFA was present in 96% of cases (192/200, left = 97, right = 95). A single TFA was present in 95.3% and double TFAs were present in 4.7% of cases. In 91.7%, the TFA originated from the superficial temporal artery, and in 3.1%, it originated from the external carotid artery. One left TFA originated from the maxillary artery. The TFA was significantly longer on the right than on the left side (56.6±26.0 versus 47.3±22.2 mm; p = 0.03). The TFA mean diameter was 1.0±0.4 mm (range: 0.4–2.2 mm) with no difference between face sides. TFA length correlated with its diameter (r = 0.46, p <0.05). The TFA always originated below the zygomatic arch, and it should be found in the 8.8 mm wide area beginning 17.0mm below the lower border of the zygomatic arch.

**Conclusions:**

The TFA has a significant role in lateral face vascularization, and absence of this vessel is very uncommon.

## Introduction

The transverse facial artery (TFA) is a branch of the superficial temporal artery that perfuses the lateral face. It is usually a small vessel, running transversely and ending in the buccal area. It lies on a masseter muscle, inferior to the lower border of the zygomatic branch of the facial nerve and superior to the parotid duct. In its proximal part, it is surrounded by parotid gland [1]. Moreover, many anastomoses with the facial, masseteric, buccinator, and the infraorbital arteries are present, which, together with the TFA, perfuse the lateral face [1,2].

Knowledge of the lateral face’s topographical anatomy, especially the vascularization of this region, is crucial in aesthetic and plastic surgery procedures. In the aesthetic surgery, a face lift flap is a common procedure, and TFA perforators are one of the most important arterial supply source for this flap [3,4] Damage to the TFA can cause reduction of blood supply to the face lift flap that may lead to surgical failure. Moreover, face lift procedures are connected with the risk of various complications such as hematoma, facial nerve palsy, and facial skin sloughing and necrosis [5]. The face flap procedure is usually performed bilaterally, which makes reasonable when comparing the TFA morphology on the left and right sides of the face [6]. Currently face transplant is more frequently used to treat patients with acquired facial defects, and the TFA is also involved in these complex procedures [7,8]. Finally, since midface injuries, including TFA injury, may also occur due to road traffic accidents or explosive incidents, this artery is also present in the area of interests of trauma surgeons [9].

Clinically, a color Doppler ultrasound is used to assess TFA anatomy and other external carotid artery branches prior to lateral face procedures, but in some cases, external carotid angiography may yield better results [10]. Despite the indubitable clinical significance of the TFA, only a few studies regarding its anatomy were performed. Nevertheless, there is a lack of complex morphological studies performed on a large sample in this topic. Therefore, the aim of the present study was to assess detailed morphometrical TFA features, which will be especially helpful for aesthetic and plastic surgeons performing procedures in the lateral face region.

## Patients and methods

### Study group

The methods and protocols were carried out in accordance with the approved guidelines by the Bioethical Committee of the Jagiellonian University, Krakow, Poland (No. 1072.6120.213.2017). The need for consent was waived by the ethics committee.

The TFA evaluation in this retrospective study was performed on patients who had had a head computed tomography angiography (CTA) in the Department of Diagnostic Imaging, Injury Centre of Emergency Medicine and Disaster, Jagiellonian University Medical College in Kraków, between January 2018 and July 2018. In total, 100 patients (57.0% females) were included in this study with bilateral TFA visualization. The average age of the included subjects was 53.6 ± 20.3 years old.

### Computed tomography angiography method and image evaluation

The CTA was performed on a multi-row computed tomography scanner (GE Optima CT 660; GE Healthcare, Chicago, IL, USA). Seventy milliliters of the nonionic contrast agent, iomeprol (Iomeron 350; 350 mg iodine/mL; Bracco Imaging, Milan, Italy), was injected. The scanning procedure was started automatically after the bolus reached the common carotid artery at the of C3-C4 level. The examined region ranged from the aortopulmonary window to the top of the head. The scanner settings were 120 kV, 200 mA, and 64x0.625-mm slice collimation. Axial 0.625 mm slices at an increment of 1.25 mm were reconstructed with a matrix of 512x512 after applying a standard kernel.

The data was analyzed using three-dimensional (3D) volume-rendering image postprocessing on an Advantage Workstation AW4.5 (GE Healthcare, Chicago, IL, USA). The volume-rendering opacity was set to values of 45 to 300 HU. Reconstructions were then investigated visually. Data for both sides of the TFA was recorded separately.

### Measurements

**Fig 1:**
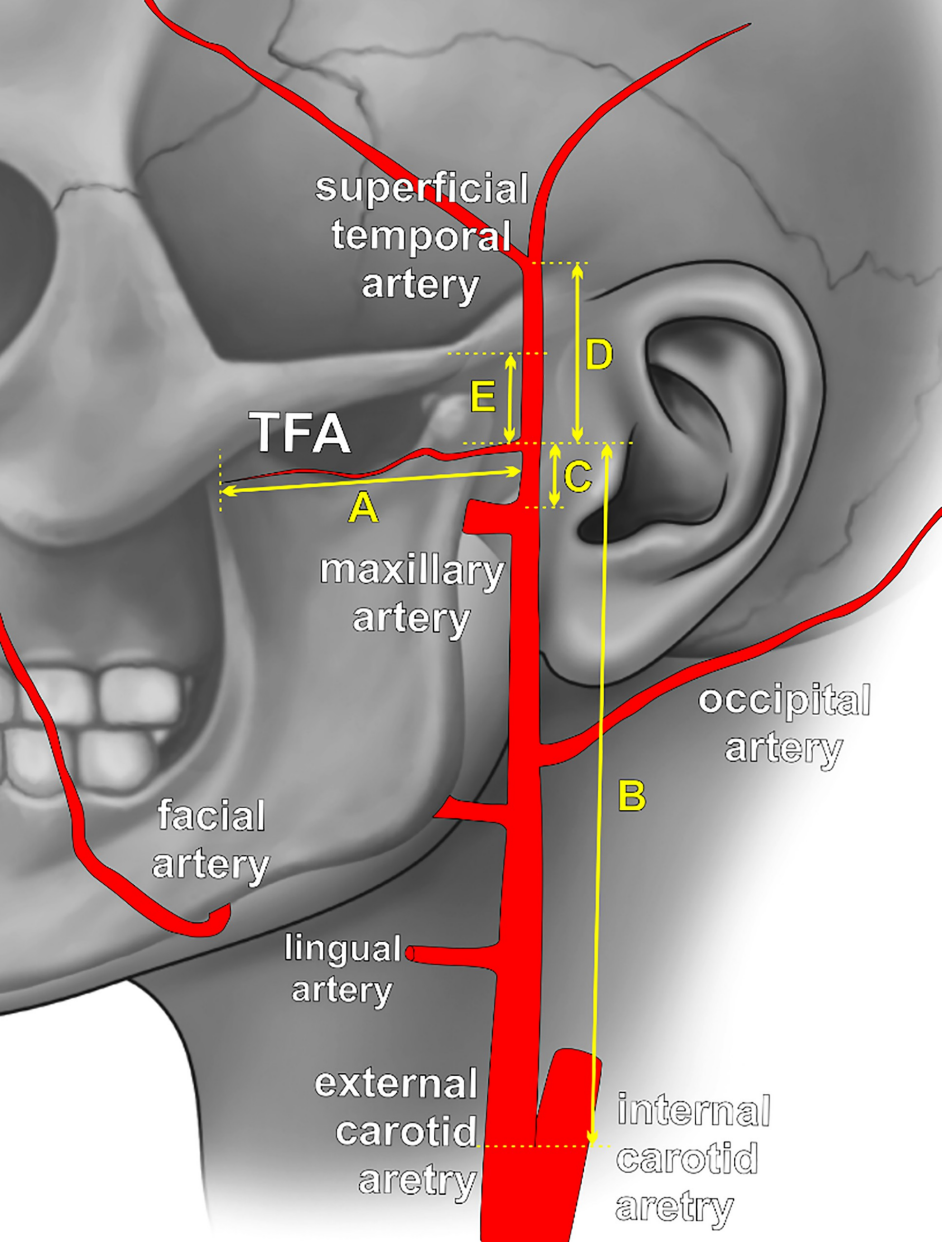
Schematic view of the external carotid artery tree with marked measurements points. A—the length of transverse facial artery (TFA), measured on its course; B—distance between the TFA origin and bifurcation of common carotid artery; C—distance between the TFA origin and bifurcation of external carotid artery; D—distance between the TFA origin and superficial temporal artery bifurcation; E—distance between the TFA origin and lower border of the zygomatic arch.

In every patient, the TFA was identified. The number of TFAs and origins were recorded. Measured parameters included several parameters (Fig 1):

- the length of TFA, measured over its course;
- the TFA diameter measured 1 cm from its origin;
- diameter of common carotid artery measured 1 cm before its bifurcation;
- diameter of maxillary artery measured 1 cm after external carotid artery bifurcation;
- diameter of superficial temporal artery measured 1 cm before its bifurcation;
- distances between TFA origin and bifurcation of both common carotid artery and external carotid artery;
- distance between TFA origin and superficial temporal artery bifurcation; and
- distance between TFA origin and lower border of the zygomatic arch.

### Statistical analysis

Qualitative features were presented by frequencies and percentages. Normal distribution was assessed with the Shapiro-Wilk test. A *p*-value of <0.05 was considered to be statistically significant. Quantitative features were characterized using the mean value ± standard deviation. To evaluate the clinical range, some distances were presented using the 10^th^ and 90^th^ percentiles. To compare TFA features between left and right sides, the paired t-test or Wilcoxon range test was used depending on whether data was normally distributed. The power analysis indicated that in order to detect a simple correlation r (r = 0.2) using a two-sided test, 5% significance level test (α = 0.05), and 80% power (β = 0.2), the required minimal sample size was approximately 193. The statistical analyses were performed with STATISTICA v13.1 (StatSoft Inc., Tulsa, OK, USA) for Windows.

## Results

### Number and origin of TFA

**Fig 2:**
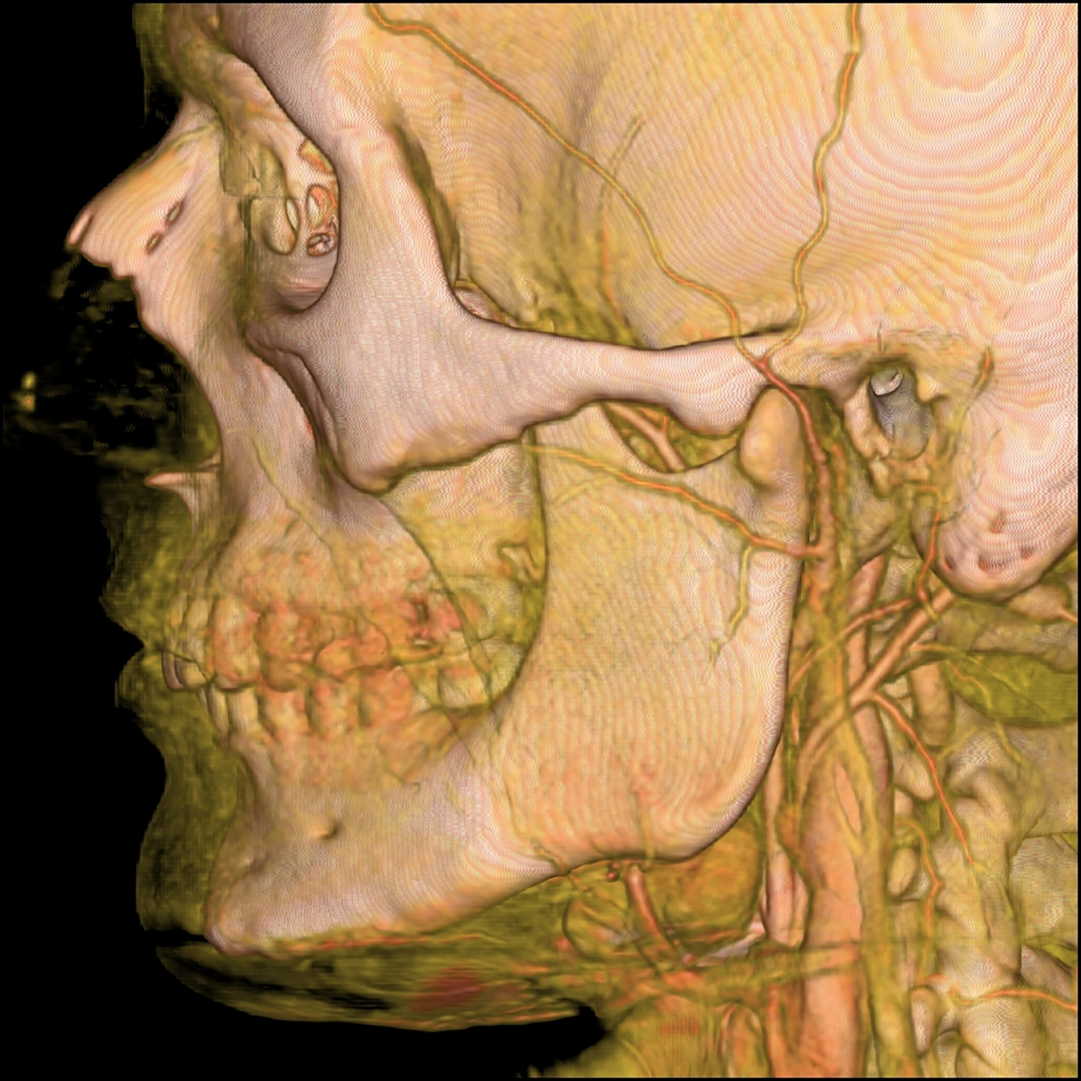
The computed tomography angiography (CTA) of the external carotid artery with three-dimensional (3D) volume-rendering showing typical single transverse facial artery (TFA) with masseteric branches. Black arrow indicates TFA.

**Fig 3:**
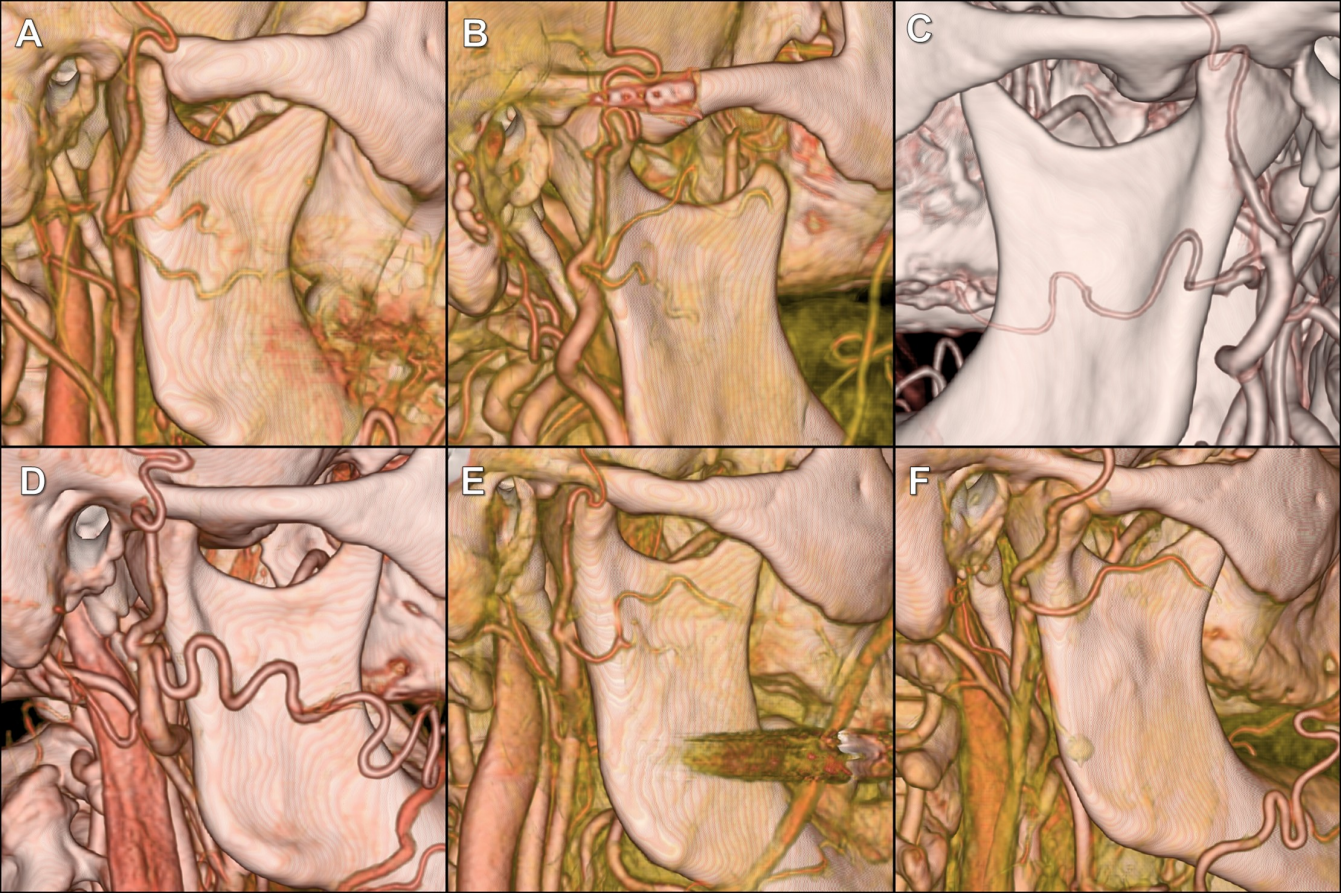
The CTAs of the external carotid artery with 3D volume-rendering showing variations of the transverse facial artery (TFA). Black arrows indicate TFA.(A) Double TFA, upper TFA is divided into another two branches. (B) Double TFA. (C) TFA branches off from the maxillary artery. (D) Three arteries arising from the same point: superficial temporal artery, maxillary artery and TFA. The latter is dominating over the facial artery, which was hypoplastic. (E and F) Three arteries arising from the same point: superficial temporal artery, maxillary artery and TFA.

The TFA was present in 96% of cases (192/200). The TFA was present on the left side in 97% (97/100) and on the right side in 95% (95/100) of all analyzed heads. A single TFA was present in 95.3% (183/192, left = 92, right = 91; Fig 2) and double TFA was present in 4.7% (9/192, left = 5, right = 4; Fig 3) of cases. In 91.7% (176/192, left = 88, right = 88), the TFA originated from the superficial temporal artery. In 3.1% (6/192, left = 4, right = 2; Fig 3), the TFA originated from the external carotid artery before its division to the superficial temporal and maxillary arteries. In 4.7% (9/192, left = 4, right = 5; Fig 3), the external carotid artery was divided into three arteries arising from the same point: (1) superficial temporal artery; (2) maxillary artery; and (3) TFA. One of those cases (0.5%) was associated with TFA that was dominant over the hypoplastic facial artery. In 0.5% (1/192, left = 1, right = 0; Fig 3), the TFA originated from the maxillary artery.

### Measurements of TFA and other structures

The morphometric TFA characteristics with a division to the left and right side are shown in Table 1. The TFA was significantly longer on the right than on the left side (56.6 ± 26.0 versus 47.3 ± 22.2 mm; p = 0.03). In 1.0% (2/192, left = 2, right = 0) the TFA was shorter than 20 mm, in 28.1% (54/192, left = 34, right = 20) shorter than 40 mm, and in 43.8% (84/192, left = 46, right = 38) it was shorter than 60 mm.

**Table 1:** The morphometric and topographic characteristics of the transverse facial artery (TFA) with division on the left and right side (mean ± standard deviation) in mm. Significant differences were marked in bold.

	Total	Right	Left	p
Length of TFA	51.9 ± 24.4	56.6 ± 26.0	47.3 ± 22.2	**0.030**
Diameter of TFA	1.0 ± 0.4	1.0 ± 0.3	1.0 ± 0.4	0.971
Diameter of the external carotid artery	3.2 ± 0.5	3.2 ± 0.5	3.2 ± 0.5	0.072
Diameter of the maxillary artery	2.0 ± 1.2	1.8 ± 0.4	2.1 ± 1.7	**0.000**
Diameter of the superficial temporal artery	1.42 ± 0.42	1.44 ± 0.40	1.41 ± 0.44	0.450
Distance from the common carotid artery bifurcation to TFA	57.5 ± 11.1	57.2 ± 11.5	57.8 ± 10.8	0.879
Distance from the external carotid artery bifurcation to TFA	2.6 ± 2.1	2.6 ± 2.0	2.5 ± 2.2	0.947
Distance from the superficial temporal artery bifurcation to TFA	39.9 ± 15.2	39.0 ± 17.3	38.8 ± 16.6	0.686
Distance from the lower border of zygomatic arch to TFA	20.9 ± 4.1	21.0 ± 4.1	20.7 ± 4.1	0.397

The TFA mean diameter was 1.0 ± 0.4 mm (range: 0.4–2.2 mm) with no differences between face sides. The artery with the diameter <1 mm was present in 49.0% (94/192, left = 52, right = 42), while the artery with the diameter >2 mm existed in 2.1% (4/192, left = 2, right = 2). The length of the TFA correlated with its diameter (r = 0.46, p <0.05).

The TFA originated near the external carotid artery bifurcation (mean: 2.6±2.1, range: 0.0–10.8mm with no differences between face sides) and was shifted closer to the superficial temporal artery bifurcation than the external carotid artery origin (Table 1). The TFA always originated below the zygomatic arch and was approximately 20.9 ± 4.1 mm (range: 6.2–47.3 mm) away from the arch. The distance between the TFA origin and zygomatic arch was positively correlated with the TFA length (r = 0.26, p = 0.011; Table 2). Using the 10th and 90th percentiles to calculate the average location of the TFA origin, the TFA should be found in the 8.8 mm wide area beginning 17.0 mm below the lower border of the zygomatic arch.

**Table 2:** Correlations of transverse facial artery (TFA) length and diameter with other measured parameters. Significant correlations are highlighted in bold print.

	Length of TFA		Diameter of TFA	
	r	p	r	p
Diameter of external carotid artery	**0.21**	**0.043**	0.11	0.271
Diameter of maxillary artery	**0.23**	**0.023**	**0.25**	**0.016**
Diameter of superficial temporal artery	**0.32**	**0.003**	**0.36**	**0.001**
Distance from common carotid artery bifurcation to TFA	-0.14	0.174	–0.13	0.195
Distance from external carotid artery bifurcation to TFA	0.08	0.423	–0.01	0.931
Distance from superficial temporal artery bifurcation to TFA	0.17	0.088	–0.04	0.676
Distance from lower border of zygomatic arch to TFA	**0.26**	**0.011**	0.08	0.429

Both length and diameter of the TFA were positively correlated with maxillary artery diameter (r = 0.2, p = 0.023 and r = 0.25, p = 0.016, respectively, Table 2) and superficial temporal artery diameter (r = 0.32, p = 0.003 and r = 0.36, p = 0.001, respectively, Table 2). Furthermore, the diameter of maxillary artery correlated with diameter of external carotid artery (r = 0.32, p <0.001).

## Discussion

Most of available data regarding TFA morphometry are based on its examination in cadaveric studies and are performed on a sample size <44 cases [3,11–14]. Moreover, the topographical anatomy of the TFA course with special regard to soft tissues surrounding it such as the parotid gland and duct, masseter muscle, or facial nerve were assessed in two cadaveric studies by Yang et al. and Lee et al. but the study populations were limited [9,12]. To our best knowledge, no studies regarding TFA morphometry using CTA of living patients were performed; however, a study by Renshaw et al. used color Doppler ultrasonography to evaluate TFA diameter and its origin [8].

The data presented in our study confirmed that TFA occurs in almost all cases. However, the number of TFA located on the same side of the face ranged from one to two. Compared to the study performed by Yang et al., there were no cases of triple TFA, and the prevalence of double TFA was much lower (4.7% versus 25.0%)[12]. This discrepancy may result from insufficient CTA resolution in which very tiny arteries may not be visible, or vessels could overlap, which would explain lower prevalence of multiple TFA variants. On the other hand, those differences could be also explained by different populations represented in studies (Caucasian versus Asian). Other TFA parameters measured in the Yang et al. study were not comparable with the current paper; however, they add valuable information to clinical practice [12]. The authors assessed the number of TFA perforators, which ranged from one to four, and have noted that most perforators penetrated the superficial cutaneous tissue perpendicularly below the duct on the anterior third of the masseter muscle [12].

The origin of the TFA in the current study was also quite constant, and the TFA originated from superficial temporal artery in 91.7% of cases. This finding is in stark contrast with the data presented by Cormack and Lamberty in which 35.0% of TFAs originated from external carotid artery or its terminal bifurcation [13]. In our study, only 3.1% of TFAs originated from external carotid artery and 4.7% from its bifurcation to superficial temporal and maxillary artery. Moreover, in the present study, one TFA originated from maxillary artery. A similar case was reported by Baçar et al [11]. Finally, our study described one case of facial artery hypoplasia connected with recompensing TFA hyperplasia. Previous reports have shown similar arterial configurations and underlined their practical significance in facial plastic surgery [15,16]. In face allotransplantation, TFA domination over hypoplastic or absence of a facial artery is an important factor. Thus, surgical planning should be associated with accurate pre-operative imaging. Renshaw et al. proposed that those procedures should be preceded by color Doppler examination to assess anatomical and also physiological domination of arteries[8]. However, in patients with reduced blood flow, external carotid artery angiography may yield better results[10]. Furthermore, our study delivers clinically significant data regarding the location of the proximal part of the TFA. We have calculated an empirical zone in which the majorities of the TFA should be found, and it extends over the temporomandibular joint from the 17^th^ to 26^th^ mm below the lower border of the zygomatic arch.

Lee et al. described topographic relations of TFA and zygomatic and buccal branch of facial nerve [9]. In their study, the vertical distance between the facial nerve zygomatic branch and TFA was very short, measuring on average 3 mm at the height of the zygion (the most lateral point of the zygomatic arch). Also, at the height of zygion, the distance between the TFA and the buccal branch of the facial nerve averaged 12.8 mm. The TFA supplies the lateral face, parotid gland, parotid duct, masseter muscle, facial nerve, and the integument [1]. Moreover, based on computed tomography scans from 40 cadaveric hemifaces, Zhao et al. found that the TFA has a great role in vascularization of the lateral orbit and malar plexus. In 23.0% of cases, the TFA was a trunk artery for lateral orbit and malar plexus, and in another 31.0%, this plexus was equally vascularized by the TFA, zygomatic-orbital artery, and premasseteric branch of the facial artery [17].

As mentioned above, the clinical anatomy of the TFA is especially important in face lift flaps [18]. The superficial musculoaponeurotic system is perfused by the transverse facial artery perforator branches on their path to the subdermal plexus; thus, the artery is at risk for injury during both elevation of the skin flap and elevation of the superficial musculoaponeurotic system [3]. The most common complication of face lift flap is a hematoma, which can even endanger the survival of the flap [9]. The most important cause of this complication is direct TFA transection [9]. In subcutaneous facelifts in which the superficial musculoaponeurotic system is not comprised in the flap, necrosis is more often observed [19]. As face lift flaps are commonly performed bilaterally, we compared TFAs morphology on both sides of the face [6]. Similar values were observed on both sides, except for the mean artery length, which was longer on the right side than on the left side. There is no clear explanation as to why significant differences occurred. In a study by Banks et al., it was observed that unilateral carotid dominance was seen in the nasal dorsum and tip in cadavers [14]. On the other hand, bilateral perfusion was seen in the maxilla. Most likely, development of the facial artery determines the range of the TFA supply.

Another role for the TFA can be seen in the superselective intra-arterial chemotherapy in advanced maxillary sinus cancer, in which the cisplatin can be administered directly to the tumor. In two out of ten patients, the TFA is a significant feeding artery of the tumor and may be used for direct infusion [20].

The current study is not without limitations. First, no correlations between the TFA course and surrounding soft tissues and the course of facial nerve were investigated. The corresponding veins were also not assessed, especially since preservation of these veins is also important in preventing complications. Second, the depth at which the TFA runs and its location in individual tissue layers could not be recorded using CTA. Third, due to the low resolution of CTA, very small vessels of the studied region could not be detected. Fourth, no interracial differences were investigated as the analyzed patients were only Caucasian. Finally, the presented morphometrical features of studied arteries should not be used as a universal tool because variations may occur in individual patients.

## Conclusion

The TFA has a great role in lateral face vascularization and absence of this vessel is very uncommon. The average length of the TFA is about 5 cm, and it is significantly longer on the right side of the face. The diameter of the artery is relatively small and is below 1 mm in about half cases. When present, the TFA should be found below the zygomatic arch in the region extending from 17^th^ mm below the zygomatic arch with a width of 8.8 mm.

Knowledge of TFA anatomy is important in avoiding any complications in plastic and craniofacial surgery in addition to patients with vascular compromise during oncological, aesthetical operations, and trauma involving the lateral face.
